# A web-based prognostic nomogram for the cancer specific survival of elderly patients with T1-T3N0M0 renal pelvic transitional cell carcinoma based on the surveillance, epidemiology, and end results database

**DOI:** 10.1186/s12894-022-01028-1

**Published:** 2022-05-24

**Authors:** Jinkui Wang, Jie Tang, Xiaozhu Liu, Dawei He

**Affiliations:** 1 Department of Urology; Ministry of Education Key Laboratory of Child Development and Disorders; National Clinical Research Center for Child Health and Disorders (Chongqing); China International Science and Technology Cooperation Base of Child Development and Critical Disorders; Chongqing Key Laboratory of Pediatrics, Children’s Hospital of Chongqing Medical University, 2 ZhongShan Rd, Chongqing, 400013 People’s Republic of China; 2 Department of Epidemiology, Public Health School, Shenyang Medical College, Shenyang, China; 3 Department of Cardiology, The Second Affiliated Hospital of Chongqing Medical University, Chongqing, China

**Keywords:** Nomogram, Elderly patients, Renal pelvic, Transitional cell carcinoma, Cancer specific survival, SEER

## Abstract

**Background:**

At present, there are few studies on renal pelvic transitional cell carcinoma (RPTCC) in elderly patients in the literature. The study aims to establish a new nomogram of cancer-specific survival (CSS) in elderly patients with T1-T3N0M0 RPTCC and validate its reliability.

**Methods:**

This study downloaded the data of 1375 elderly patients with T1-T3N0M0 RPTCC in the Surveillance, Epidemiology, and Final Results (SEER) database from 2004 to 2018. Patients were randomly divided into training cohort (n = 977) and validation cohort (n = 398). Proportional subdistribution hazard analyse was applied to determine independent prognostic factors. Based on these factors, we constructed a compting risk model nomogram. We use the calibration plots, the area under the receiver operating characteristics curve (AUC), concordance index (C-index), and decision curve analysis (DCA) to validate predictive performance and clinical applicability. Patients were divided into low-risk group and high-risk group based on nomogram risk score. Kaplan–Meier curve was applied to analyze the difference in survival curve between the two groups of patients.

**Results:**

We found that the risk factors affecting CSS in elderly patients with T1-T3N0M0 RPTCC are surgery, AJCC stage, laterality, tumor size, histological grade, and tumour laterality. Based on these factors, we established a nomogram to predict the CSS of RPTCC patients at 1-, 3-, and 5-year. The calibration plots showed that the predicted value was highly consistent with the observed value. In the training cohort and validation cohort, the C-index of the nomogram were 0.671(95% CI 0.622–0.72) and 0.679(95% CI 0.608–0.750), respectively, the AUC showed similar results. The DCA suggests that namogram performs better than the AJCC stage system. The Kaplan–Meier curve showed that CSS of patients was significantly higher in the low-risk group.

**Conclusions:**

In this study, the SEER database was used for the first time to create and validate a new nomogram prediction model for elderly patients with T1-T3N0M0 RPTCC. Compared with the traditional AJCC stage system, our new nomogram can more accurately predict the CSS of elderly patients with T1-T3N0M0 RPTCC, which is helpful for patient prognosis assessment and treatment strategies selection.

**Supplementary Information:**

The online version contains supplementary material available at 10.1186/s12894-022-01028-1.

## Introduction

Urothelial carcinoma (UC) is one of the most common malignant tumors in the world, and its incidence is the fourth [1]. It can also be called transitional cell carcinoma(TCC) of the urinary tract. It occurs in the transitional cells of the urinary tract, and the onset includes the lower urinary tract (bladder, urethra) and upper urinary tract(renal pelvis, ureter). Although UC is a high-incidence cancer, 95% of urothelial cancers are bladder cancer [2], while upper urinary tract urothelial carcinoma (UTUC) only accounts for 5–10% [3]. In recent years, the morbidity and mortality of UTUC have gradually increased [3, 4]. UTUC includes renal pelvic transitional cell carcinoma(RPTCC) and ureter transitional cell carcinoma, RPTCC is the most common pathological type of renal pelvis cancer, accounting for all 5–7% of the total incidence of kidney tumors, and the incidence is about 2–3 times higher than that of ureter transitional cell carcinoma [4]. RPTCC is more common in men and the elderly [5], especially those between 70 and 90 years old, the average age of diagnosis is 73 years [6]. Due to the extremely thin muscular layer of the renal pelvis, 60% of RPTCC have infiltrated outside the kidney at the time of diagnosis [6]. In addition, the absence or neglect of symptoms in early stages also be one of the reasons that RPTCC infiltrates to the kidney outside. Therefore, the overall prognosis of RPTCC is poor, and the 5-year survival rate is less than 50% [7]. Nephroureterectomy and bladder cuff resection is the most common treatment for RPTCC, and only a small number of patients undergo partial nephroureterectomy or local resection [8], it is only used for tumors with low-grade, low-stage, and tumor size less than 2 cm [9].

Most clinicians and medical researchers use the American Joint Committee on Cancer(AJCC) stage system to stage malignant tumors, which is a standard system widely used in tumor treatment evaluation and prognosis evaluation worldwide. However, the AJCC stage system lacks clinical pathological factors that may have an important impact on the prognosis of RPTCC, such as age, gender, surgical method, histological grade, marriage, etc. [10, 11]. Survival prediction models for elderly patients with RPTCC are still lacking, and there is an urgent need for a reliable and accurate prognostic model.

Nomogram is a simple graphical mathematical model that can predict the occurrence of a given event by generating a single numerical estimate based on specific clinical and pathological variables [12–14]. Nomogram has been widely used in clinical decision-making for various tumors, including liver cancer, breast cancer, lung cancer and so on [9, 15, 16]. As far as we know, no nomogram model for predicting the prognosis of elderly patients with RPTCC has been established using the Surveillance Epidemiology and End Results (SEER) database. Combined with the clinical pathological parameters extracted from the SEER database, we designed a nomogram model to predict the prognosis of elderly patients with T1-T3N0M0 RPTCC. This model can predict the clinical outcome of the patient and provide help for the clinical decision-making of patients and doctors.

## Methods

### Data source and data extraction

We extracted raw clinical data from the National Cancer Institute's Surveillance, Epidemiology, and End Results (SEER) Program to identify patients over 65 years of age in the United States from 2004 to 2018 who were diagnosed with AJCC stage I to III RPTCC. The SEER database contains 18 population-based tumor registries and covers about 28% of Americans. The data analyzed in this study is available on SEER (http://seer.cancer.gov/) [9]. Due to the demographic information, tumor characteristics, and survival status of patients in the SEER database are anonymously disclosed, our research does not require patient consent or ethical review. Our research methods comply with the regulations and rules of the SEER database.

**Fig. 1:**
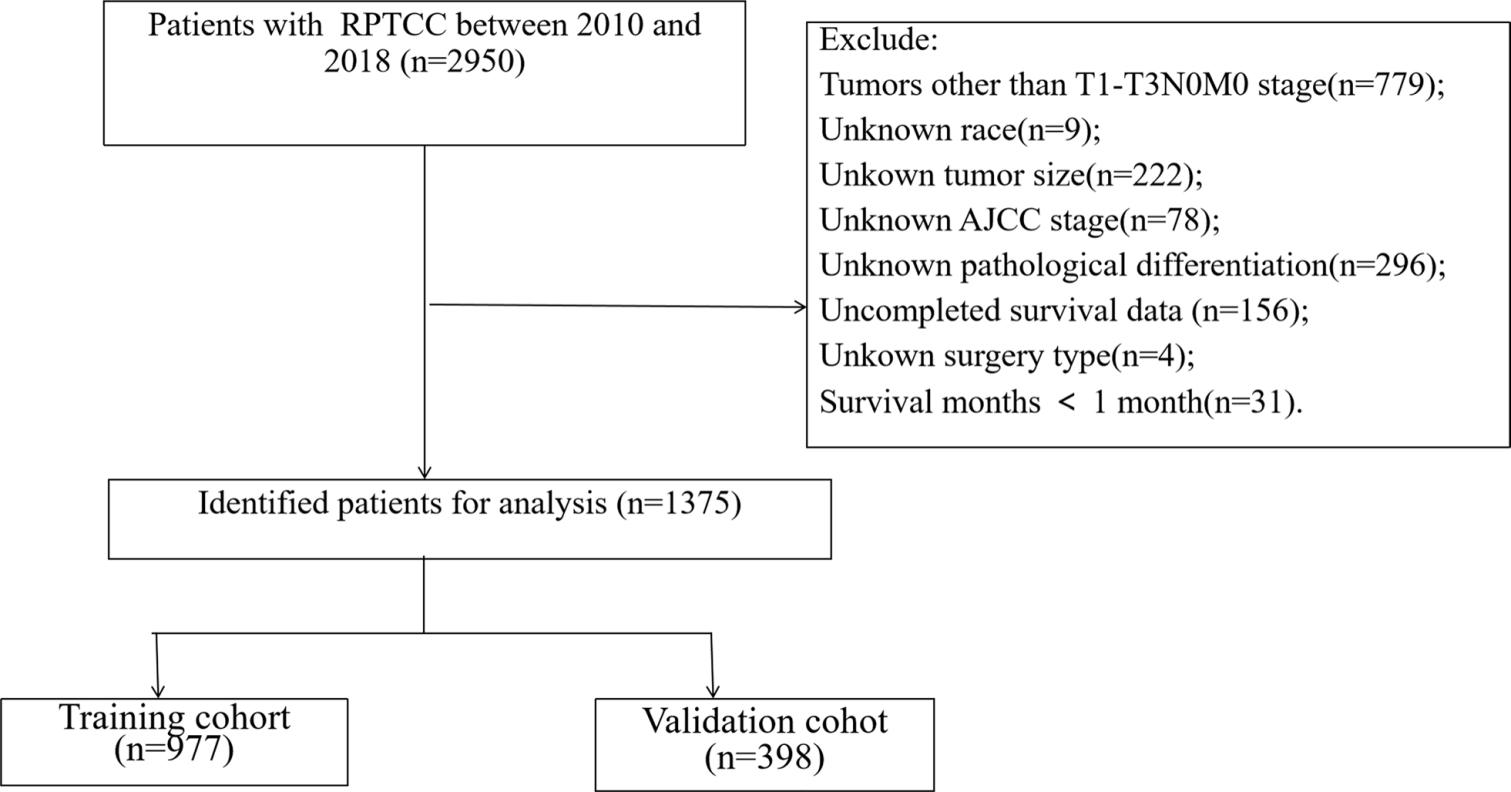
The flowchart of including and dividing patients

After excluding unknown or missing clinical pathological information, a total of 1375 elderly patients with T1-T3N0M0 RPTCC were included. We collected clinical pathological information including age at diagnosis, gender, race, tumor laterality, AJCC stage, histological type, histological grade, surgery type, chemotherapy, survival time, marital status and other. The selection criteria are: (1) age ≥ 65 years; (2) the International Classification of Diseases for Oncology, third edition (ICD-O-3) categories, code 8120, 8330; (3) T1-T3, N0, M0; (4)Unilateral RPTCC. The exclusion criteria are: (1) unknown race; (2) unknown tumor size; (3) unknown AJCC stage; (4) uncompleted survival data; (5) unknown surgery type; (6)Unknown pathological differentiation; (7)survival time < 1 month (patients who survived less than one month were more likely to die not from cancer but from other causes, such as complications related to surgery). The flowchart for selecting patients is shown in Fig. 1.

The tumor histological grade is divided into four grades (I: well-differentiated; II: moderately differ entiated; III: poorly differentiated; IV: undifferentiated or anaplastic). The race includes white, black, and other races (American Indian/AK Native, Asian/Pacific Islander). The AJCC stage system we used was based on the Derived AJCC Stage Group. The size of the tumors were divided into three categories: 2, 2–4, and > 4 cm [17]. The surgical methods were divided into four groups according to the SEER Renal Surgery Code 2018, including non-surgical group, local tumor excision group, partial nephrectomy group and radical nephrectomy group. The outcome of these patients in the study was death caused by RPTCC.

### Nomogram construction and validation

We collected a total of 1375 patients, who were randomly divided into training cohort 70% (n = 977) and validation cohort 30% (n = 398). Proportional subdistribution hazard analyse used to determine independent risk factors for RPTCC in elderly patients, and calculated the hazard ratio (HR) and 95% confidence interval (CI). We incorporated these important parameters into the compting risk model nomograph to predict the 1-year, 3-year, and 5-year CSS rate of elderly patients with RPTCC. The reliability and accuracy of the predictive model were evaluated by an internal validation cohort. We constructed the area under the curve (AUC) and calculated the C-index to reflect the prediction accuracy and discriminability of the nomogram. We constructed 1000 bootstrap resamples and used the calibration plots to validate the nomogram.

### Clinical utility

We constructed a clinical decision curve analysis (DCA) to assess the clinical significance of our nomogram [18]. It is a new algorithm that evaluates the clinical utility value of nomogram by estimating the net benefit under each risk threshold. Also, we divide patients into low-risk group and high-risk group based on the cut-off value of the nomogram risk value. The survival rate of the two groups of patients was compared by Kaplan–Meier (K-M) curve and log-rank test.

### Statistical analysis

All statistical analysis used R software (version 3.4.1; http://www.Rproject.org). and SPSS software (version 23.0, SPSS, Chicago, IL, USA). R software package "cmprsk", "DynNom", "nomogramformula", "survival", "survivalROC", "RMS", "ggDCA", "survminer", "shiny", "foreign" were used to construct and validate the nomogram model, and ROC curve and DCA were constructed to validate the nomogram model. The difference between risk groups was confirmed by log-rank test to confirm the significant difference. P values less than 0.05 indicated that the results were statistically significant.

## Results

### Clinical features

We included a total of 1375 elderly patients with RPTCC. The clinical pathological characteristics of all patients as well as the training cohort and the validation cohort are shown in Table 1. Among them, the distribution of age at each stage was relatively balanced. There were more men (56.1%), most of them were white (87.6%), and the majority were married (58.1%). Among the tumor-related features, the most AJCC stage was stage III (48.1%, followed by stage I (37.5%) and stage II (14.5%). The histological grade was mostly grade III/IV (84.1%). Most of the tumor size was 2-4 cm (48.9%), followed by more than 4 cm (34.8%), and the tumor was slightly more on the left side (51.3%). Most patients had received radical nephrectomy (94.0%), less people received chemotherapy (13.4%). The mean survival time of all patients was 47.3 ± 39.4 months, and the median survival time was 37 (inter-quartile range 16–69) months. There was no significant difference between the training cohort and the validation cohort.

**Table 1:** Clinicopathological characteristics of patients with T1-T3N0M0 RPTCC

	Total (N = 1375)	Training cohort (N = 977)	Validation cohort (N = 398)	p
Age				0.355
65–69	267 (19.4%)	182 (18.6%)	85 (21.4%)	
70–74	297 (21.6%)	205 (21.0%)	92 (23.1%)	
75–79	308 (22.4%)	231 (23.6%)	77 (19.3%)	
80–84	290 (21.1%)	210 (21.5%)	80 (20.1%)	
> 84	213 (15.5%)	149 (15.3%)	64 (16.1%)	
Race				0.048
White	1204 (87.6%)	862 (88.2%)	342 (85.9%)	
Black	55 (4.00%)	43 (4.40%)	12 (3.02%)	
Other^a^	116 (8.44%)	72 (7.37%)	44 (11.1%)	
Sex				0.996
Male	772 (56.1%)	548 (56.1%)	224 (56.3%)	
Female	603 (43.9%)	429 (43.9%)	174 (43.7%)	
Year of diagnosis				0.540
2004–2008	381 (27.7%)	275 (28.1%)	106 (26.6%)	
2009–2013	484 (35.2%)	335 (34.3%)	149 (37.4%)	
2014–2018	510 (37.1%)	367 (37.6%)	143 (35.9%)	
Laterality				0.864
Left	705 (51.3%)	499 (51.1%)	206 (51.8%)	
Right	670 (48.7%)	478 (48.9%)	192 (48.2%)	
AJCC stage				0.710
I	515 (37.5%)	372 (38.1%)	143 (35.9%)	
II	199 (14.5%)	142 (14.5%)	57 (14.3%)	
III	661 (48.1%)	463 (47.4%)	198 (49.7%)	
Marriage				0.414
No	576 (41.9%)	402 (41.1%)	174 (43.7%)	
Yes	799 (58.1%)	575 (58.9%)	224 (56.3%)	
Grade				0.580
I/II	218 (15.9%)	151 (15.5%)	67 (16.8%)	
III/IV	1157 (84.1%)	826 (84.5%)	331 (83.2%)	
Surgery				0.844
No Surgery	23 (1.67%)	17 (1.74%)	6 (1.51%)	
Local tumor excision	29 (2.11%)	19 (1.94%)	10 (2.51%)	
Partial nephrectomy	30 (2.18%)	20 (2.05%)	10 (2.51%)	
Radical nephrectomy	1293 (94.0%)	921 (94.3%)	372 (93.5%)	
Radiotherapy				0.610
No	1356 (98.6%)	965 (98.8%)	391 (98.2%)	
Yes	19 (1.38%)	12 (1.23%)	7 (1.76%)	
Chemotherapy				0.276
No	1191 (86.6%)	853 (87.3%)	338 (84.9%)	
Yes	184 (13.4%)	124 (12.7%)	60 (15.1%)	
Tumor size				0.233
< 2 cm	225 (16.4%)	150 (15.4%)	75 (18.8%)	
2–4 cm	672 (48.9%)	488 (49.9%)	184 (46.2%)	
> 4 cm	478 (34.8%)	339 (34.7%)	139 (34.9%)	
Survival months (mean (SD))	47.3 (39.4)	47.1 (39.6)	47.6 (39.0)	0.851
Survival months (median (IQR))	37 (16, 69)	36(16, 69)	38.5 (16, 69)	0.705
Status				0.480
Alive	622 (45.2%)	452 (46.3%)	170 (42.7%)	
Cancer cause of death	426 (31.0%)	296 (30.3%)	130 (32.7%)	
Other cause of death	327 (23.8%)	229 (23.4%)	98 (24.6%)	

^a^American Indian/AK Native, Asian/Pacific Islander

*AJCC* American Joint Council on Cancer; *IQR* inter-quartile range

### Proportional subdistribution hazard analysis

We incorporated parameters into the proportional subdistribution hazard analysis, including age, race, sex, marriage, AJCC stage, histological grade, tumor size, laterality, surgery type, radiotherapy, and chemotherapy. The results of analysis showed that variables such as age, laterality, histological grade, AJCC stage, surgery type, tumor size, marriage were statistically significant, suggesting that these variables were independent risk factors and can effectively predict CSS in elderly patients with RPTCC. In addition, risk factors for death from other causes were analyzed. We found that age, year of diagnosis, and surgery were significant factors for mortality from other causes. The results of the proportional subdistribution hazard analysis are shown in Table 2.

**Table 2:** Proportional subdistribution hazard analysis in training cohort

	Cancer-specific mortality			Other causes mortality		
	HR	95% CI	P	HR	95% CI	P
Age						
65–69	Reference			Reference		
70–74	0.99	0.67–1.46	0.95	1.18	0.68–2.05	0.56
75–79	1.07	0.74–1.55	0.72	2.49	1.54–4.01	< 0.001
80–84	1.25	0.85–1.83	0.25	2.93	1.81–4.74	< 0.001
> 84	1.40	0.93–2.12	0.11	3.31	2–5.48	< 0.001
Race						
White	Reference			Reference		
Black	1.16	0.65–2.07	0.61	0.92	0.43–1.98	0.84
Other	1.21	0.79–1.86	0.38	0.74	0.45–1.21	0.23
Sex						
Male	Reference			Reference		
Female	1.12	0.87–1.45	0.38	0.82	0.63–1.07	0.14
Year of diagnosis						
2004–2008	Reference			Reference		
2009–2013	1.12	0.85–1.46	0.42	0.68	0.52–0.89	0.005
2014–2018	0.85	0.62–1.18	0.33	0.39	0.25–0.6	< 0.001
Laterality						
Left	Reference			Reference		
Right	1.38	1.09–1.75	0.006	0.83	0.64–1.06	0.14
AJCC stage						
I	Reference			Reference		
II	1.41	0.95–2.08	0.087	1.33	0.93–1.88	0.11
III	2.26	1.72–2.98	< 0.001	0.83	0.61–1.13	0.23
Marriage						
No	Reference			Reference		
Yes	0.82	0.63–1.07	0.14	0.89	0.68–1.16	0.38
Grade						
I/II	Reference			Reference		
III/IV	1.85	1.24–2.75	0.002	0.80	0.59–1.07	0.13
Surgery						
No Surgery	Reference			Reference		
Local tumor excision	1.41	0.52–3.86	0.5	0.83	0.19–3.54	0.8
Partial nephrectomy	0.23	0.06–0.81	0.022	2.04	0.69–6.05	0.2
Radical nephrectomy	0.42	0.18–0.96	0.039	1.00	0.42–2.38	< 0.001
Radiotherapy						
No	Reference			Reference		
Yes	3.35	1.6–7.02	0.001	0.54	0.14–2.03	0.36
Chemotherapy						
No	Reference			Reference		
Yes	1.05	0.74–1.49	0.78	0.61	0.37–1.03	0.063
Tumor size						
< 2 cm	Reference		0.78	Reference		
2–4 cm	1.31	0.9–1.9	0.16	1.28	0.87–1.89	0.22
> 4 cm	1.58	1.07–2.33	0.02	1.14	0.76–1.73	0.35

*HR* hazard ratio; *CI* confidence interval

### Nomogram construction for 1-, 3-, and 5-year CSS

**Fig. 2:**
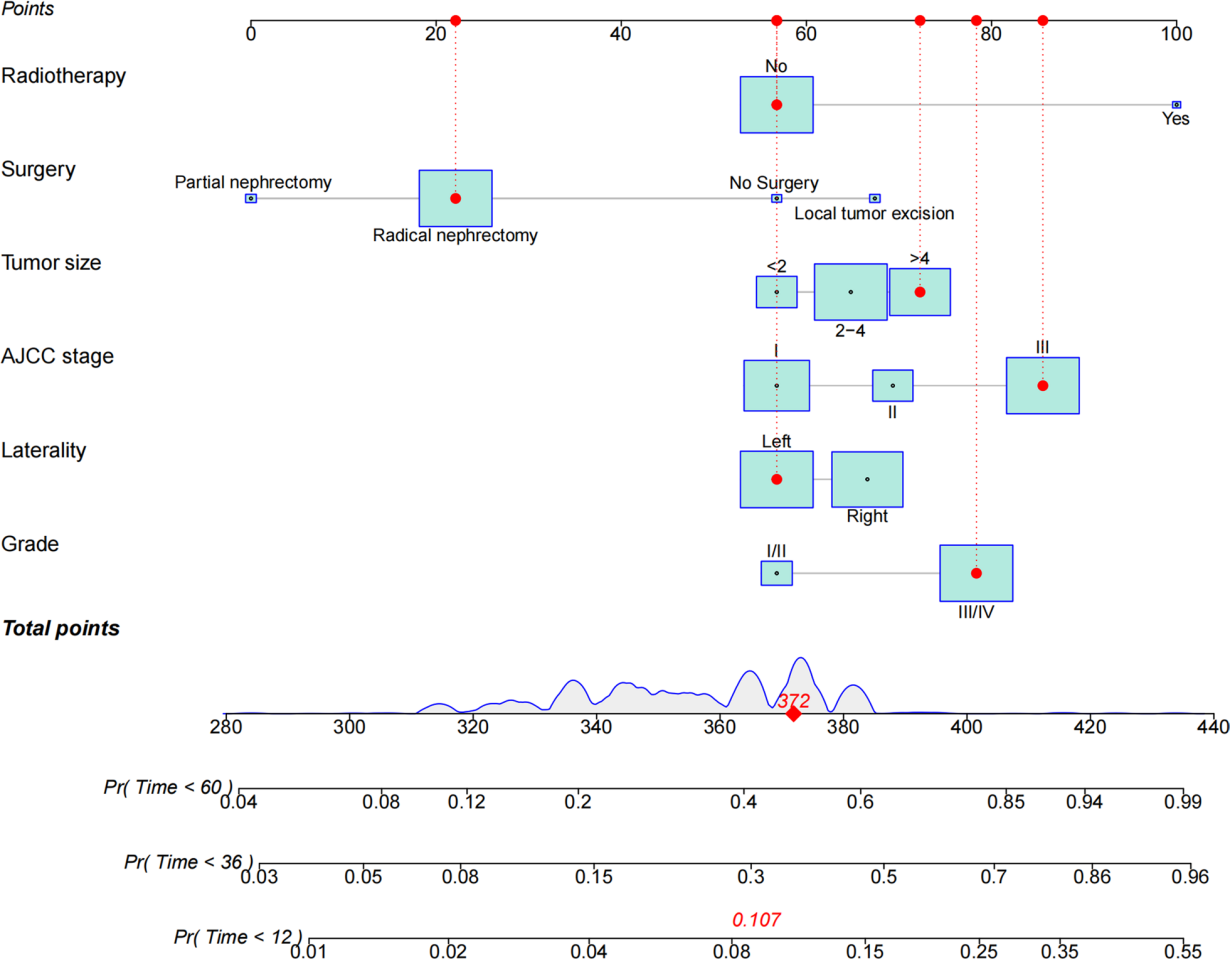
Nomogram for 1-, 3-, and 5-year CSS of elderly patients with T1-T3N0M0 RPTCC;

We created a new nomogram using variables determined by proportional subdistribution hazard analysis to predict 1-, 3-, and 5-year CSS in elderly patients with RPTCC (Fig. 2). It can be seen from this model that surgery has the greatest impact on CSS of patients, followed by AJCC stage, and other variables such as laterality, tumor size, histological grade, and radiotherapy have similar effects.

### Validation of the nomogram

**Fig. 3:**
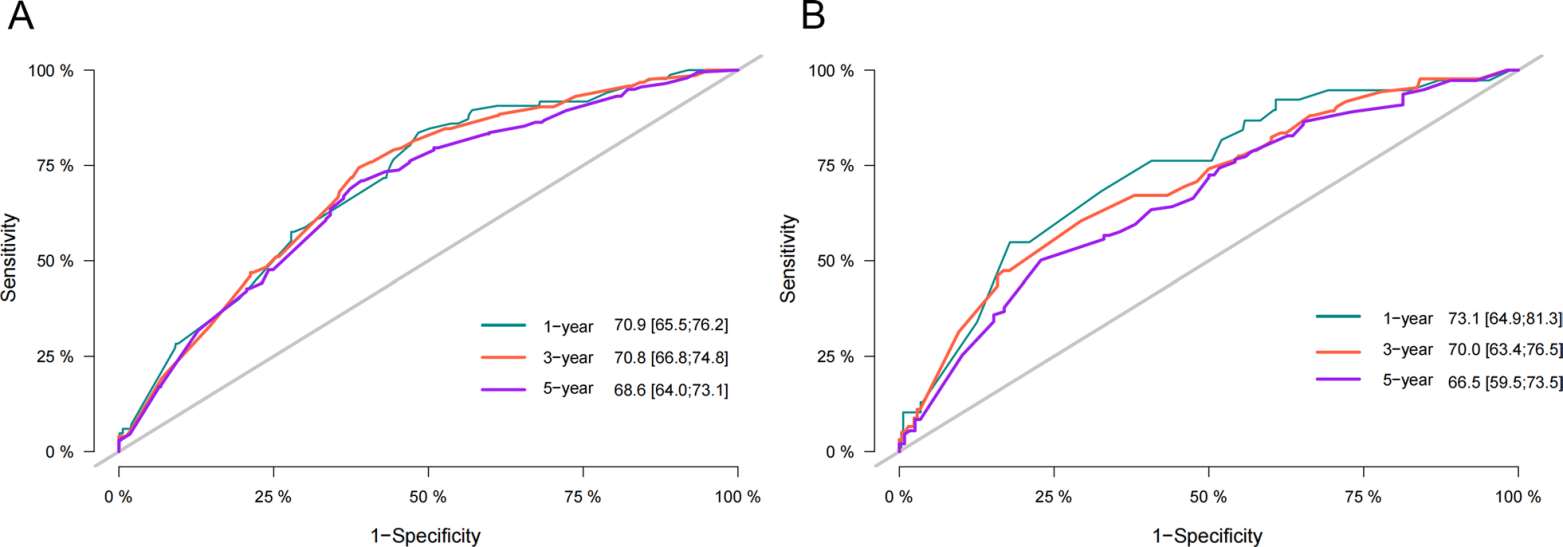
The AUC of 1-, 3- and 5-year of training cohort (**A**) and validation cohort (**B**)

**Fig. 4:**
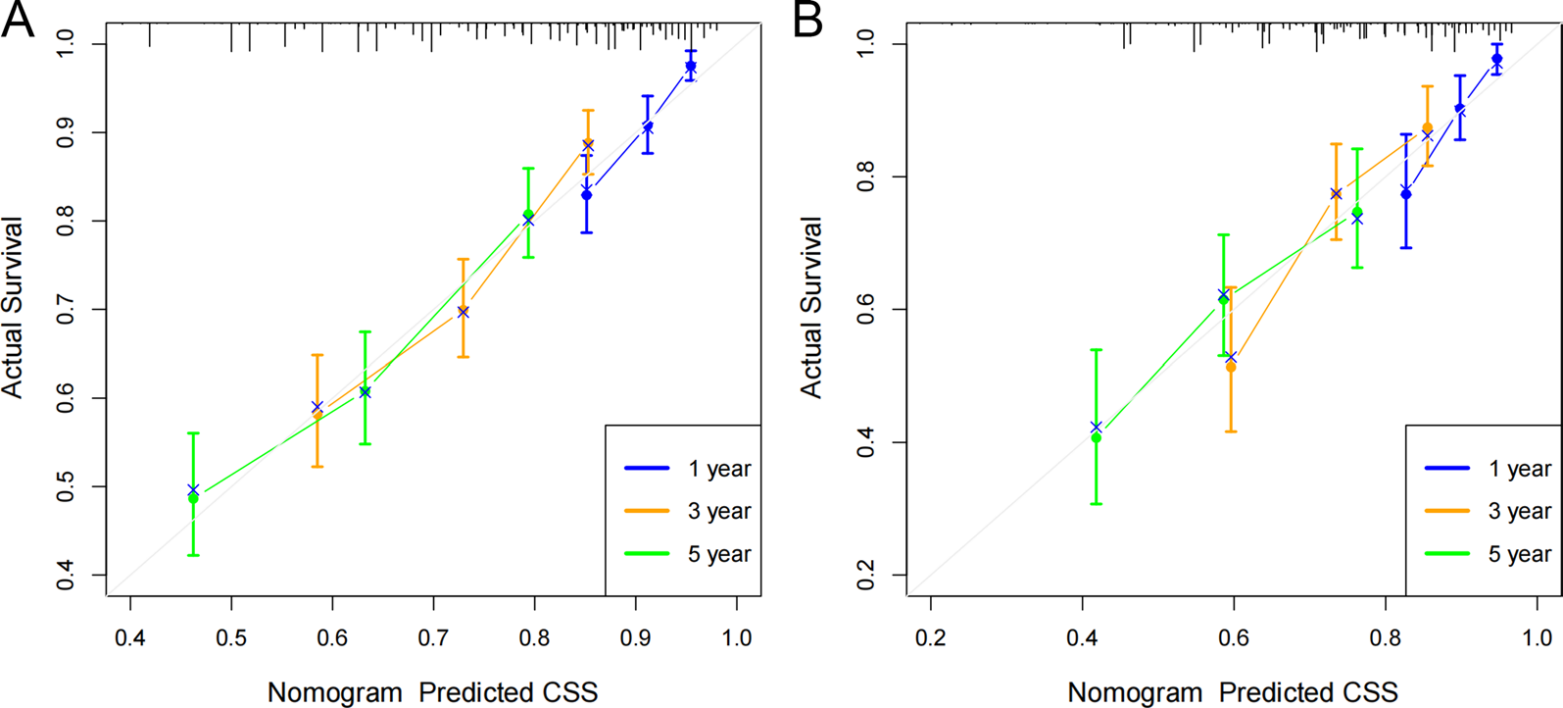
Calibration plots of nomogram. **A** For 1-, 3-, and 5-year CSS in training cohort; **B** For 1-, 3-, and 5-year CSS in validation cohort

The C-index of the nomogram was higher than that of the AJCC stage, which are the training cohort [0.671 (95% CI 0.622–0.72) vs. 0.617 (95% CI 0.584–0.650)] and the validation cohort [0.679 (95% CI 0.608–0.750) vs. 0.591 (95% CI 0.530–0.652)]. Further analysis of the ROC curve, similar to the C-index results, the 1-, 3-, and 5-year AUC values of the training cohort were 0.709, 0.708, and 0.686, respectively; and the 1-, 3-, and 5-year AUC values of the validation cohort were 0.731, 0.700, 0.665, respectively (Fig. 3A–B). The calibration plots indicated that the 1-, 3-, and 5-year CSS probability standard curves of the nomogram model were close to the diagonal, and the predicted value was highly consistent with the observed value, indicating that our namogram calibration ability was good(Fig. 4A–B).

### Clinical application of the nomogram

**Fig. 5:**
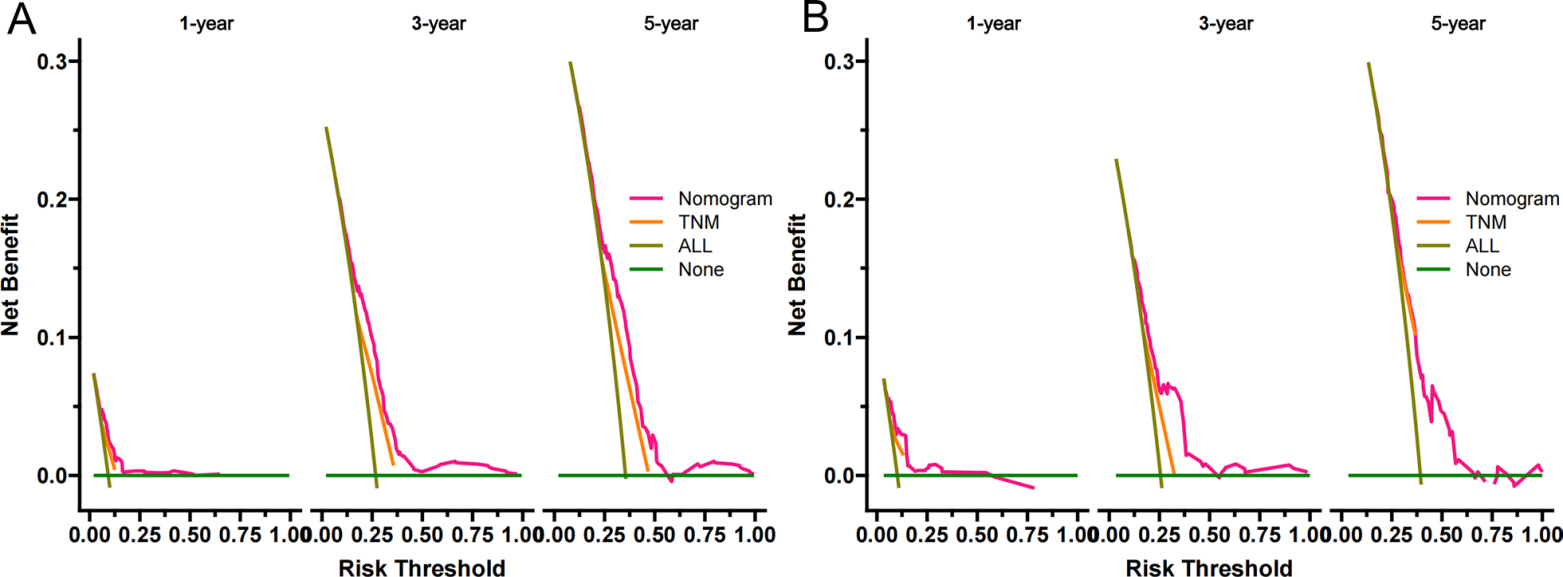
Decision curves of the nomogram predicting CSS in the training cohort (**A**) and validation cohort (**B**). The x-axis is the threshold probability, and the y-axis is the net benefit. The green line indicates that no patients have died, and the dark green line indicates that all patients have died. When the threshold probability is between 15 and 50%, the net benefit of the model exceeds all deaths or no deaths

**Fig. 6:**
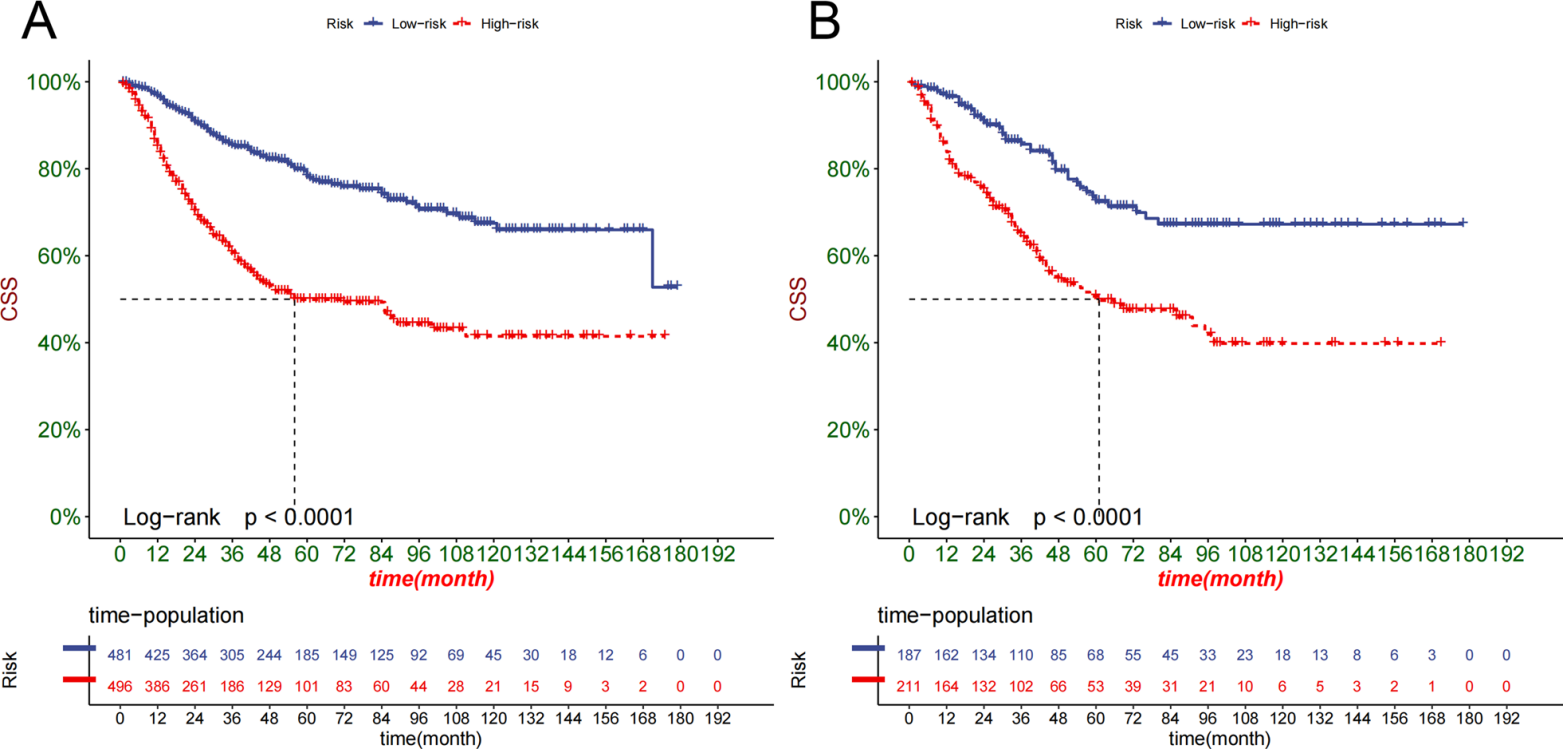
Kaplan–Meier curves of CSS for patients in the low- and high-risk groups in the training cohort (**A**) and validation cohort (**B**)

The DCA curve showed that the predictive ability of namogram for 1-, 3- and 5-year in the training and validation cohorts was better than the AJCC stage system (Fig. 5A–B). Based on the total score of patients on the nomogram, we developed a risk stratification system. These patients were divided into low-risk group (total score ≤ 95.9) and high-risk group (total score > 95.9). In the training cohort and validation cohort, the Kaplan–Meier curve indicated that the CSS of patients in the low-risk group was higher than that in the high-risk group (Fig. 6A–B).

### Online application for CSS prediction

We had developed a user-friendly online application for CSS prediction of elderly patients with T1-T3N0M0 RPTCC based on the nomogram model, which can be accessed at https://jinkuiwangnomogram1.shinyapps.io/DynNomapp/. The estimated CSS probability can be obtained immediately after inputting patient characteristics, this online prediction tool is convenient to use in clinical practice.

## Discussion

This study used a large number of clinical samples to create a nomogram prediction model based on the SEER database to predict the mid- and long-term prognosis of elderly patients with RPTCC. Although the AJCC stage system was commonly used clinically to evaluate the prognosis of tumors and the choice of treatment strategies, nomogram survival prognosis prediction model has higher accuracy and practicability than the traditional AJCC stage system [19, 20]. A study showed that the morbidity and mortality of RPTCC are on the rise, and it is more important to accurately predict the prognostic survival rate of RPTCC [21]. RPTCC is common in elderly patients with a poor prognosis, but compared with metastatic RPTCC, patients with RPTCC have a higher survival rate [6]. At present, there is a lack of survival prediction model for elderly RPTCC. The high mortality rate of RPTCC in the elderly made it necessary to develop a namogram prediction model, which can provide doctors and patients with accurate prognostic predictions and treatment options. In this study, we constructed a nomogram and validated its accuracy and practicability based on the SEER database. We used multivariate Cox regression analysis to screen the variables that affect the CSS of patient, including age at diagnosis, surgery, AJCC stage, laterality, tumor size, histological grade, and marriage.

Similar to previous studies, age was a risk factor for other causes mortality, and older age means lower survival rate [22]. However, marriage was not a factor in cancer-specific deaths or deaths from other causes in patients. Although previous studies have shown that married patients may receive more financial support and mental comfort, which is conducive to the survival of patients [23]. And there was a study showing that marriage is associated with a low risk of cancer metastasis [24]. We did not find a significant effect of gender on CSS.

In addition, we found that laterality was also a factor affecting patient survival. In order to eliminate the influence of confounding factors, we performed inverse probability of treatment weighting matching analysis for tumor laterality, and the results still suggested that tumor laterality was a factor affecting patient survival (Additional file 1: Fig. S1). Laterality has not been reported to influence survival in patients with RPTCC. Previous study have proved that lymph node dissection is a factor affecting the survival of patients with RPTCC [25]. As a result, It is possible that anatomical differences (e. g. the presence of the rather vulnerable abdominal vena cava on the right side) may had an impact on the amount of lymph nodes resected on each side thereby potentially influencing CSS.

The characteristics of the tumor itself were also important factors affecting the survival rate of patients, including tumor size, AJCC stage, and histological grade. Among them, it can be seen from the nomogram that AJCC stage was the biggest risk factor. The CSS of patients with AJCC stage III tumors was significantly lower than that of patients with stage I (HR 2.384, 95%CI 1.796–3.165). Secondly, the tumor grade was also an obvious influencing factor. Poorly differentiated and undifferentiated or anaplastic were independent risk factors for CSS of patients, due to these tumors were more aggressive and lead to a worse prognosis. Tumor size has always been an critical factor influencing the prognosis of patients with kidney tumors. Our study was the same as the previous study [26], patients with larger tumor mean shorter survival time. The standard method of treatment for renal pelvic cancer is surgical treatment, and in our study, radical nephrectomy was the surgical method for most patients. Radical nephrectomy includes full length resection of the kidney and ureter, bladder sleeve resection, and regional lymph node dissection.

The survival rate of patients with partial nephrectomy and radical nephrectomy was significantly higher than that of patients with local tumor excision and those who did not undergo surgery, as can be seen from the nomogram. To test whether patients who underwent partial nephrectomy had a higher survival rate than those who underwent radical nephrectomy. We used inverse probability of treatment weighting matching analysis to exclude the influence of other variables, and then used K-M curve and log-rank test to test the difference in survival between the two groups. The results showed that there was no significant difference in survival between the two groups (Additional file 1: Fig. S2). The results indicate that radical nephrectomy is still the preferred treatment for patients with RPTCC, and partial nephrectomy can also be used for patients who cannot tolerate radical nephrectomy.

We used these selected prognostic factors to establish a predictive model for the prognosis of elderly patients with RPTCC. We compared the namogram model with the traditional AJCC stage system, the C-index and AUC were both higher in namogram model, indicating that the model was superior to the traditional AJCC stage system. And we used the calibration plots to validate the predictive ability of the training cohort and the validation cohort, the results showed that our prediction model has calibration abilities and good discrimination [27]. It can be seen from DCA that the nomogram prediction model can accurately predict the CSS of elderly patients with RPTCC at 1-, 3-, and 5-year, which is better than the AJCC stage system. We also used a risk stratification system to divide patients into high- and low-risk group. We found that the survival rate of patients in the high-risk group was significantly lower. It showed that our prediction model can accurately identify high-risk patients, which is of great significance to the formulation of treatment strategies and follow-up evaluation for high-risk group patients. The variables used to construct the nomogram in this study are easy to obtain in clinical practice and are friendly to both patients and doctors.

However, our research still had some limitations. First of all, this study is a retrospective study based on the SEER database, and there may be selection bias. Second, some important life indicators, such as smoking, drinking, and BMI index are not available in the SEER database, so the model is not perfect [28, 29]. However, according to previous research findings [30], we had included vital parameters such as age, surgery type, AJCC stage, tumor size and histological grade. Third, surgical details are an important factor affecting patient survival, such as the approach (e. g. open vs. laparoscopic vs. robotic surgery) and the extent of resection (e. g. the proportion of patients in which the ureter has been entirely removed to cases with only partial resection of the ureter). Unfortunately, we can't get enough surgical details from the database. In the future, more detailed surgical details could increase the accuracy of the prediction model. Fourth, no data on comorbidities are incorporated in this study, comorbidities frequently hamper the ability to undergo various treatment options especially in elderly patients. Finally, we only used the data of the SEER database for internal validation, independent external validation and even prospective trials validation are necessary for our model.

## Conclusion

This study successfully used clinical pathological parameters to construct a namogram prediction model to predict the survival and prognosis of elderly patients with T1-T3N0M0 RPTCC. The model had been validated and confirmed that it can accurately predict the 1-, 3-, and 5-year CSS of elderly patients. It can help doctors and patients choose treatment and follow-up strategies. However, there are still many limitations to our predictive model. Further external validation and the addition of variables are key to improving model performance.

## Supplementary Information

**Additional file 1.** Propensity score matching analysis.

## Data Availability

The data analyzed in this study is available at https://seer.Cancer.gov/.

## Abbreviations

RPTCC: Renal pelvic transitional cell carcinoma
CSS: Cancer-specific survival
UC: Urothelial carcinoma
UTUC: Upper-tract urothelial carcinoma
AJCC: American joint committee on cancer
SEER: Surveillance, epidemiology, and end results
C-index: Concordance index
AUC: Area under the receiver operating characteristic curve
DCA: Decision curve analysis
