# Supplementary material for: A web-based prognostic nomogram for the cancer specific survival of elderly patients with T1-T3N0M0 renal pelvic transitional cell carcinoma based on the surveillance, epidemiology, and end results database
Source: BMC Urol. 2022 May 24;22:78. doi: 10.1186/s12894-022-01028-1 (PMC9131540; doi:10.1186/s12894-022-01028-1)
Supplement: Supplementary file 1 — Additional file 1. Propensity score matching analysis. [file 12894_2022_1028_MOESM1_ESM.docx]

**Supplementary Material**

**A web-based prognostic nomogram for the cancer specific survival of elderly patients with** **T1-T3N0M0 renal pelvic transitional cell carcinoma based on the Surveillance, Epidemiology, and End Results Database**

**Figure S1.** Inverse probability of treatment weighting matching analysis for tumor laterality(A), K-M curves of patients with different laterality after matching(B).

**
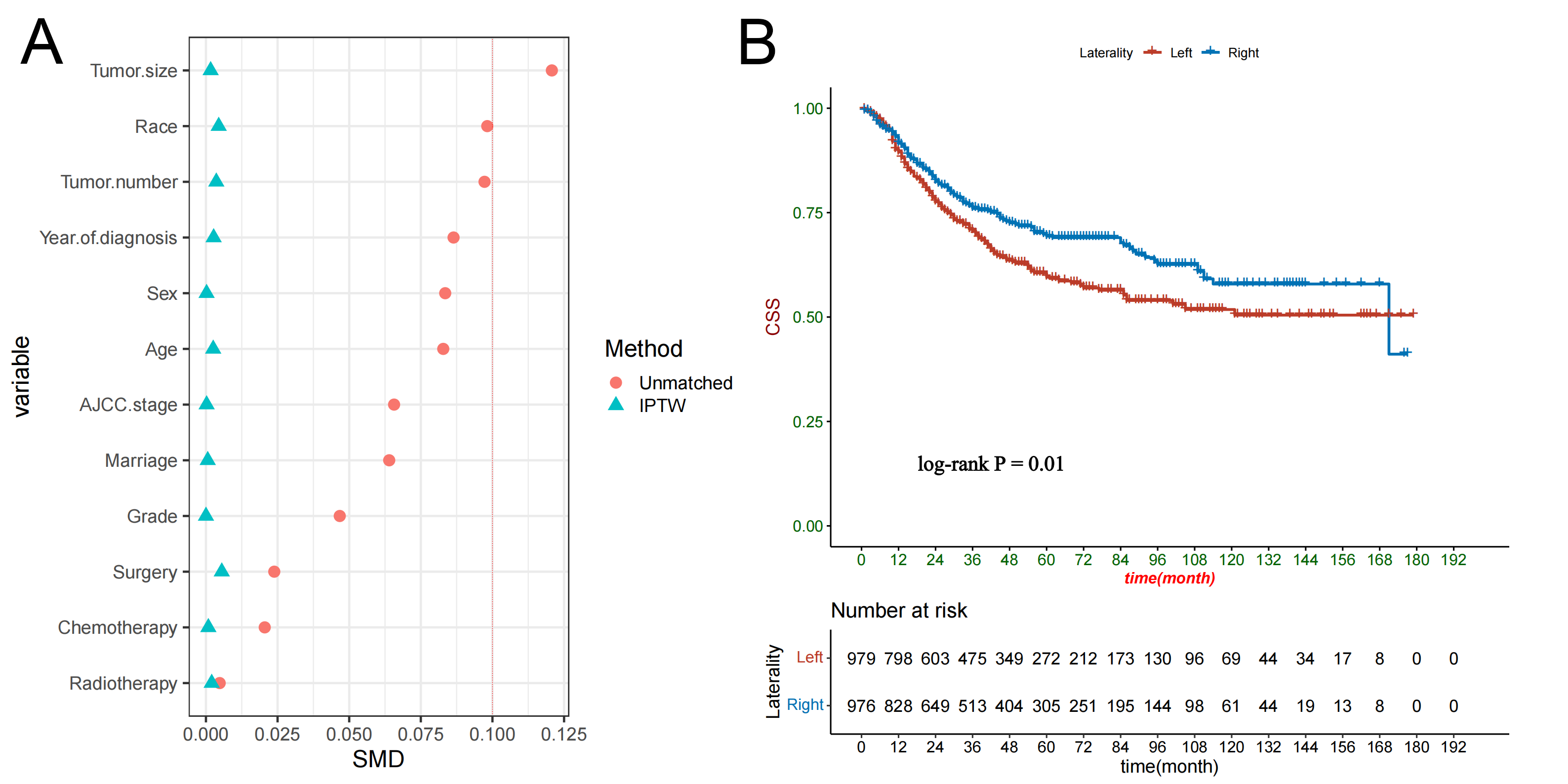
**

**Figure S2.** Inverse probability of treatment weighting matching analysis for surgery(A), K-M curves of patients with different surgery after matching(B).

**
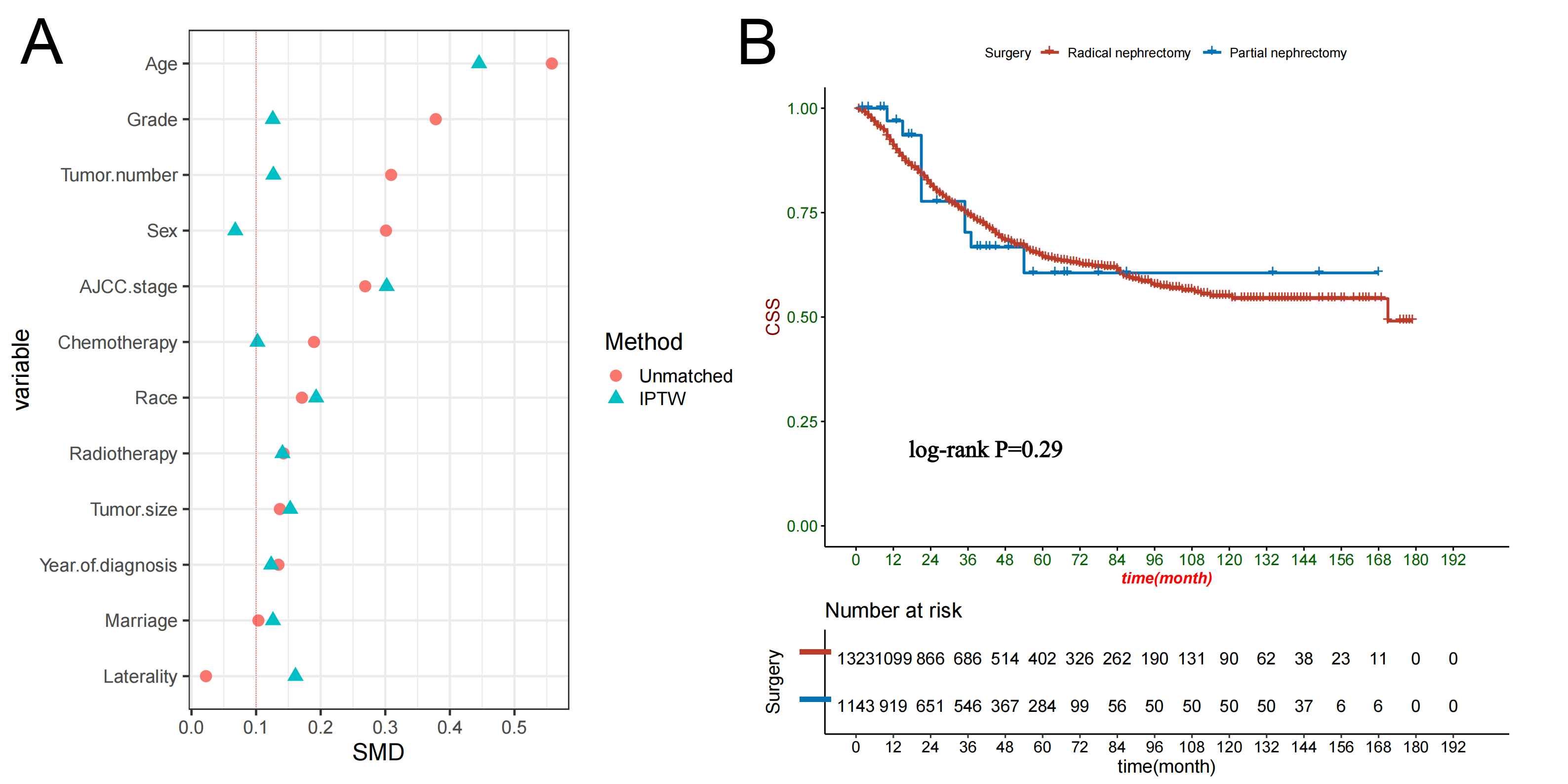
**
